# A subtelomeric region affects telomerase-negative replicative senescence in *Saccharomyces cerevisiae*

**DOI:** 10.1038/s41598-018-38000-9

**Published:** 2019-02-12

**Authors:** Pascale Jolivet, Kamar Serhal, Marco Graf, Stephan Eberhard, Zhou Xu, Brian Luke, Maria Teresa Teixeira

**Affiliations:** 1 Sorbonne Université, PSL, CNRS, UMR8226, Institut de Biologie Physico-Chimique, Laboratoire de Biologie Moléculaire et Cellulaire des Eucaryotes, F-75005 Paris, France; 2 Institute of Neurobiology and Developmental Biology, JGU Mainz, Ackermannweg 4, 55128 Mainz, Germany; 3 Institute of Molecular Biology (IMB), 55128 Mainz, Germany; 4 Sorbonne Université, PSL, CNRS, UMR7141, Institut de Biologie Physico-Chimique, Laboratoire de Physiologie Moléculaire et Membranaire du Chloroplaste, F-75005 Paris, France; 5 Present Address: Institut de Génétique Humaine, CNRS, Université Montpellier, Montpellier, France

## Abstract

In eukaryotes, telomeres determine cell proliferation potential by triggering replicative senescence in the absence of telomerase. In *Saccharomyces cerevisiae*, senescence is mainly dictated by the first telomere that reaches a critically short length, activating a DNA-damage-like response. How the corresponding signaling is modulated by the telomeric structure and context is largely unknown. Here we investigated how subtelomeric elements of the shortest telomere in a telomerase-negative cell influence the onset of senescence. We found that a 15 kb truncation of the 7L subtelomere widely used in studies of telomere biology affects cell growth when combined with telomerase inactivation. This effect is likely not explained by (i) elimination of sequence homology at chromosome ends that would compromise homology-directed DNA repair mechanisms; (ii) elimination of the conserved subtelomeric X-element; (iii) elimination of a gene that would become essential in the absence of telomerase; and (iv) heterochromatinization of inner genes, causing the silencing of an essential gene in replicative senescent cells. This works contributes to better delineate subtelomere functions and their impact on telomere biology.

## Introduction

The ends of linear chromosomes, the telomeres, are special nucleoprotein regions that are essential for the stable maintenance of chromosomes. In eukaryotes, telomeres are composed of TG-rich repeats running from the 5′ to the 3′ end of the DNA strand. The semi-conservative DNA replication of telomeres results in loss of telomeric sequences^1,2^. Telomerase, a reverse transcriptase, provides the major specific activity that counteracts this sequence loss^3,4^. Telomerase extends the 3′ protruding ends of chromosomes by reverse transcribing the repeat-containing template region of a tightly associated RNA moiety in an iterative fashion. In the absence of telomerase, telomeres shorten with each passage of the replication fork, leading to replicative senescence, characterized by a permanent cell cycle arrest, despite the cells being metabolically viable^5^. Telomere shortening is considered to be a potent tumor-suppressor, whereby in contrast, telomere maintenance mechanisms are consistently activated in cancer cells and are essential for their unlimited proliferation potential^6^. The favored current model is that in mammalian cells, as telomeres shorten progressively, they eventually lose their capacity to be concealed from the DNA damage checkpoint and activate the ATM and ATR kinases, leading to cell cycle arrest^7^.

*Saccharomyces cerevisiae* is a unicellular eukaryote that relies on telomerase activity for long-term viability^8^. Following experimental inactivation of telomerase activity, yeast cells arrest in the G2/M phase of the cell cycle after 60 to 80 population doublings. Similar to mammalian cells, this arrest depends on activation of the DNA damage checkpoint, through Mec1, the ATR orthologue in *S. cerevisiae*^9–11^. It has been reported that in cultured mammalian cells five dysfunctional telomeres are required to trigger senescence, while in telomerase negative budding yeast, a single critically short telomere is sufficient to establish cell cycle arrest^12–15^. As shortened telomeres reach a critical length, DNA repair activities such as 5′-to-3′ resection mediated by Sae2, Mre11-Rad50-Xrs2 (MRX) and Exo1 occur, resulting in the exposure of both telomeric and subtelomeric ssDNA and hence activation of Mec1^16,17^. The senescence rate should thus depend on the vulnerability of the telomeric structure at the shortest telomere to DNA repair activities and/or on its signaling capacity. Accordingly, telomere-signaling capacity can be modulated by chromatin state in the context of acute telomere dysfunction^18,19^, but whether this applies to gradual shortening in the absence of telomerase is not known.

While the genome of *S. cerevisiae* is compact - with a high gene density - the 20–30 kb regions just preceding telomeres, the subtelomeres, have fewer protein-coding genes, and no genes essential for growth in normal laboratory conditions^20^. Instead, small open reading frames with no known function accumulate in these regions. High copy gene families, such as *PAU* or *COS* are also found at subtelomeres, as well as elements with a high degree of similarity^21^. For instance, the large and short Y’ elements are 7 or 5 kb regions found at between 1 to 4 copies in nearly half of all subtelomeres. The X elements, containing a core region of about 475 bp, are present at all chromosome ends. It comprises an origin of replication, as well as some binding sites for transcription factors. The function of these elements has been elusive, due to their redundancy and only a few studies have addressed the role of these elements on the adjacent telomere *in cis*^22–24^.

In this work, we asked whether the subtelomere of the shortest telomere in the telomerase-negative cell affects replicative senescence. By comparing strains in which the pre-determined shortest telomere either harbors natural subtelomeric elements or lacks these elements, we show that removal of a terminal 15 kb subtelomeric region at the 7L chromosome end accelerates the onset of replicative senescence. This effect does not depend on the removal of the subtelomere occurring specifically at the shortest telomere in the cell. Likewise, the impact of subtelomere deletion is likely not due to impaired homology-directed repair or the absence of the conserved subtelomeric X-element, nor is it due to an identified genetic element present in the subtelomeric region. We also found that heterochromatinization of more centromere proximal genes was not the cause of poor growth. Our results contribute to an understanding of the role of subtelomeric regions with respect to rates of replicative senescence.

## Results

### A 15 kb region of the chromosome 7L subtelomere promotes viability in the absence of telomerase

**Figure 1:**
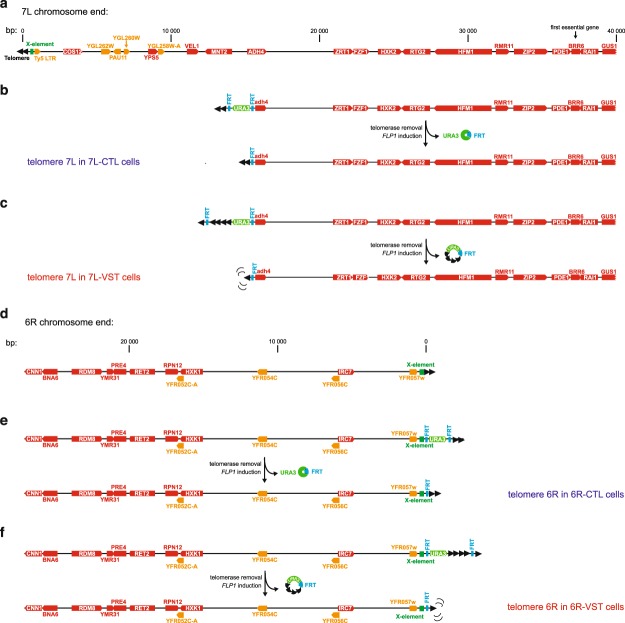
Experimental system to shorten a single telomere in the cell. The chromosome end containing the 7L telomere (**a**) is modified in two ways. In control (CTL) cells (**b**), the last 15 kb of the telomere end are replaced by a construct in which a *URA3* marker is flanked by two Flippase Recognition Target (FRT) sites and followed by a wild-type length telomeric tract. (**c**) In cells able to generate a very short telomere (VST), the *URA3* marker is followed by extratelomeric repeats that inhibit the action of telomerase on telomeric repeats *in cis*. This results in a short terminal telomeric tract. (**b**,**c**) Upon telomerase removal and induction of flippase (encoded by *FLP1*), sequences between the FRT sites are excised in a circle that is diluted out upon successive cell divisions. Remaining telomeres are of wild-type length (**b**) or very short (**c**). (**d**) The wild type 6R chromosome end is modified to generate 6R-CTL (**e**) and 6R-VST (**f**) in a manner similar to 7L (**a**–**c**). Chromosome maps and annotations are from the Saccharomyces Genome Database and Centre for Genetic Architecture of Complex Traits website (https://www.le.ac.uk/colleges/medbiopsych/research/gact/resources/yeast-telomeres) using the sequence of strain S288C as reference. Red and orange colors correspond to genes encoding minimally characterized proteins or dubious/putative proteins, respectively. Note that subtelomeric sequences of strains W303 – used in this work - and S288C – used as reference - 7L diverge in a region spanning *Ty5* to *YGL262W*.

We previously showed that senescence can be initiated by a single, experimentally derived, critically short telomere^12,13,17^. In these studies, we took advantage of a system in which we tracked a defined artificial telomere that can be shortened experimentally in a regulated manner^12,25^. This telomere is a modified version of telomere 7L (left arm of chromosome 7), in which the last 15 kb are removed, and is thus deleted for the corresponding subtelomeric elements (Fig. 1a–c). When modified (Fig. 1b,c), this telomere contains a *URA3* gene and extra-telomeric repeats flanked by two FRT sites, followed by a either a normal length (CTL), or short (VST) terminal telomeric tract. The extra-telomeric repeats inhibit telomerase action in *cis*, leading to the maintenance of the short terminal telomeric tracts (Fig. 1c). The excision of the FRT sites occurs by an inducible site-directed recombinase (Flp1, whose expression is under the control of a galactose-inducible promoter), and the corresponding loss of the *URA3* marker allows the tracking of the excision reaction.

To investigate the effects of subtelomeric elements on the signaling of a short telomere, we compared the 7L shortening system, which lacks a subtelomere, with a similar shortening system inserted at the 6R telomere with its native subtelomere still present, called 6R-VST^26^ (Fig. 1d–f). As controls, strains bearing similar constructs in which a normal-length telomere was generated following the flippase reaction, were analyzed in parallel (7L-CTL and 6R-CTL, Fig. 1b and e, respectively). In the presence of telomerase, the four sets of strains had similar growth rates (Supplementary Fig. 1a). Upon recombinase/flipase-induced telomere shortening, telomeres reached a length of about 120 bp at both 7L- and 6R –VST (Supplementary Fig. 1b). Taking into account the distribution of telomere lengths within a cell^14^, each of these modified telomeres is likely the shortest telomere in their respective strains.

**Figure 2:**
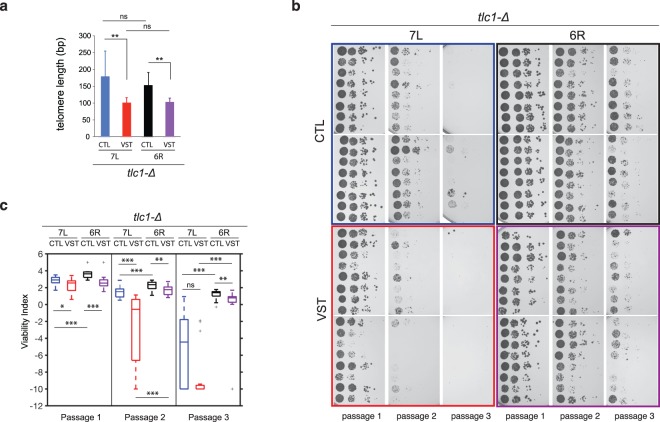
Effect of the subtelomeric region on replicative senescence. 16 telomerase-negative individual spores carrying the telomere 7L-CTL (blue), 7L-VST (red), 6R-CTL (black) or 6R-VST (purple) (see Fig. 1b,c, e,f) were germinated for two days on selective media. Colonies grown on selective plates for 2 days were then resuspended to equal concentrations and 10-fold dilutions were spotted on solid media, grown at 30 °C for 2 days (passage 1). This procedure was repeated twice (passage 2 and 3). (**a**) Cells from passage 1 were used to prepare DNA and telomere length measurements were performed by telomere-PCR using specific primers amplifying either the 7L or the 6R-derived telomeres. Median telomere length is shown. Error bars correspond to SD. Adjusted p-values were obtained by the Wilcoxon rank-sum test with a false discovery rate correction **p < 0.01 (n = 14, 14, 16 and 9, respectively). Plates were scanned at high resolution (**b**) and analyzed to obtain a numerical value for each serial dilution set that is related to the intensity of the spots (**c**). Adjusted p-values were obtained by the Wilcoxon rank-sum test with a false discovery rate correction *p-value < 0.05, **p-value < 0.01 and ***p-value < 0,001. n = 16 for 7L-CTL, 6R-CTL and 6R-VST, n = 15 for 7L-VST. See Supplementary Table 3 for detailed p-values.

We then proceeded to study the effect of the absence of the subtelomeric elements at the shortest telomere on replicative senescence in telomerase-negative cells. To this aim, we generated independent diploid strains for the 7L and 6R telomeric variants, in which the telomeric constructs are homozygous. Additionally, one allele of the telomerase RNA template-encoding gene, *TLC1*, is deleted and replaced by a nourseothricin resistance gene under the control of a promoter active only in haploid *Mat*α cells (*tlc1::Pr*α-*NatR*)^12^. After sporulation and generation of meiotic haploid progeny, we induced telomere shortening in *tlc1-Δ* cells (VST cells) by adding galactose and plating the cells on nourseothricin-containing media. 7L-CTL and 6R-CTL control strains were treated similarly. After verification of the loss of the *URA3* marker and telomere length determination, sixteen individual telomerase-negative colonies for each set of strains were assayed for their viability through 3 consecutive passages^17^ (Fig. 2a,b). Subsequent quantitative analysis of the spot assays (from Fig. 2b) measured the ability to form colonies and loss of growth potential (Fig. 2c, compare passages 1 to 3). We found that both the 7L-VST and the 6R-VST *tlc1-Δ* strains accelerated senescence compared to 7L-CTL and 6R-CTL *tlc1-Δ* strains, respectively, as previously reported^12,26^. This demonstrates that even in a native subtelomeric context, a single short telomere can induce senescence.

However, we found that the overall cell proliferation capacity differed depending on the strain used. Both the 6R-CTL and 6R-VST *tlc1-****Δ*** cells (with native subtelomeres) displayed greater proliferation potential in comparison to 7L-CTL and 7L-VST *tlc1-****Δ*** cells (lacking 7L subtelomeric elements). This suggests that natural subtelomeric elements not only have the capacity to buffer senescence onset when a critically short telomere arises, but also, that the 15 kb at subtelomere 7L is required for optimal cell growth in the absence of telomerase. Altogether, these results suggest that genetic elements present in the 7L subtelomeric region are essential for the viability of telomerase-negative cells, whether the 7L telomere is the shortest in the cell or not.

### Recruitment of homology-directed repair factors to the shortest telomere is independent of the presence of subtelomeric elements

**Figure 3:**
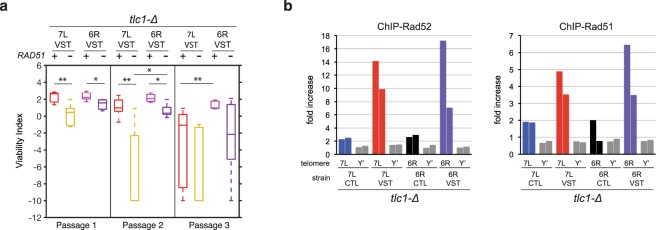
The homologous recombination machinery counteracts senescence both in the presence and absence of subtelomeric elements at the VST. (**a**) The absence of Rad51 accelerates replicative senescence in the presence or absence of subtelomeric elements at the shortest telomere. Quantitative analysis of serial spot assays of senescence for cells carrying 7L-VST or 6R-VST as in Fig. 2c. Adjusted p-values were obtained by the Wilcoxon rank-sum test with a false discovery rate correction *p-value < 0.05, **p-value < 0.01 and ***p-value < 0,001. n = 7, 6, 5, 5 for each strain set respectively. See Supplementary Table 3 for detailed p-values. (**b**) Rad51 and Rad52 preferentially associate with short telomeres in the presence and absence of subtelomere. For each indicated strain, 7L-CTL, 7L-VST, 6R-CTL or 6R-VST, a mixed population of hundreds of independent telomerase-negative clones was grown for ~30 population doublings after sporulation. Chromatin was immunoprecipitated using primary antibodies against either Rad52 (left panel) or Rad51 (right panel). The association of each protein to 7L, 6R or Y’ telomeres or to the *ARO1* locus was quantified by qPCR and the fold increase of telomere enrichment over *ARO1* is represented. Two biologically independent experiments are shown.

A major modulator of senescence is the homology dependent repair machinery, which preferentially associates with the shortest telomere in senescent cells, even before the appearance of post-senescent survivors^16,17,27,28^. Homologous recombination may promote telomere lengthening or at least limit telomere shortening to sustain cell viability in the absence of telomerase. Possible mechanisms involve sister-chromatid exchanges or inter-telomeric recombination events. Such events are expected to depend not only on the partial homology present within the degenerated telomeric repeats, but also on subtelomeric elements, which share homology among various chromosome ends. This could, in theory, account for the increased viability in 6R-VST strains compared to 7L-VST strains in that the former bears a homology region with other telomeres (through the subtelomere) while the latter does not, and hence would have an increased propensity to engage in inter-telomeric homology directed repair. If this were true, then impairing homology-directed repair should eliminate the differences in senescence between the 7L-VST and 6R-VST strains. We thus deleted Rad51 in our system and checked for the effects on proliferation capacity in telomerase-negative cells. Removal of the Rad51 recombinase resulted in a decrease in viability for all strain sets (Fig. 3a). However, the decreased cell proliferation potential of 7L-VST compared to 6R-VST strains was maintained even in the absence of Rad51, suggesting that subtelomeric elements and Rad51 act independently to sustain cell viability in the absence of telomerase.

To verify this, we asked whether the recombination factors Rad51 and Rad52 localize at the 7L- or 6R-VST telomeres to the same extent. Telomerase-negative 7L- and 6R-CTL cells, as well as 7L- and 6R-VST strains, were grown and chromatin was immunoprecipitated using primary antibodies directed against Rad51 and Rad52 (Fig. 3b). The amount of precipitated 7L-VST, 6R-VST, 7L-CTL and 6R-CTL subtelomeres were quantified by qPCR. Y’ subtelomeres were used as control. While Y’ telomeres were immunoprecipitated with the same efficiency in all strains, the modified telomeres were specifically enriched upon shortening, irrespective of the presence of subtelomeric elements. This indicates that both factors were similarly recruited to telomeres when they become critically short, suggesting that absence of subtelomeric elements in the strains containing the modified 7L chromosome end did not inhibit or promote the recruitment of repair factors.

Taken together, we concluded that Rad51 and Rad52 are recruited to the shortest telomere in senescent cells, but their role in maintaining cell viability, as well as their recruitment, is independent of subtelomeric elements.

### The 15 kb deletion of subtelomere 7L is unique in affecting cell growth in the absence of telomerase

**Figure 4:**
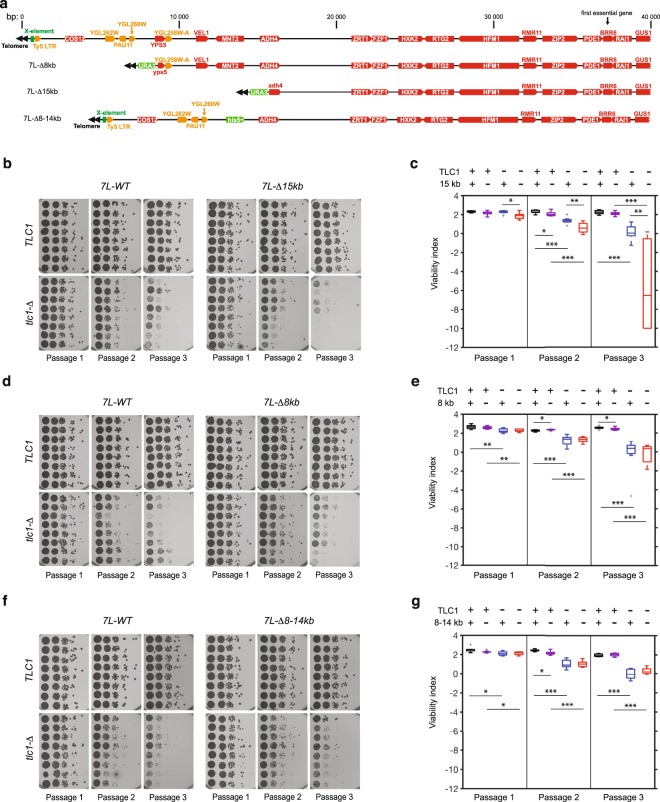
Subtelomeres affect senescence independently of the X-element. (**a**) Scheme of the deletion of the15 kb, the terminal 8 kb, and the internal 8–14 kb of the sub-telomere 7L. Annotations are as in Fig. 1a. (**b**,**d**,**f**) Spores of the indicated genotype were spotted in parallel for three serial passages and analyzed as in Fig. 2b,c (**c**,**e**,**g**). Adjusted P-values were obtained by the Wilcoxon rank-sum test with a false discovery rate correction *p-value < 0.05, **p-value < 0.01 and ***p-value < 0,001. n = 8 for each strain set. See Supplementary Table 3 for detailed p-values.

The observation that replicative senescence was accelerated in the strains lacking the subtelomeric elements even in the control length telomere (7L-CTL, compared to 6R-CTL, Fig. 2c) suggests that these two parameters – absence of subtelomere and critically short telomere – are probably independent. In other words, the lack of subtelomeric elements must act *in trans*, independently from being carried by the shortest telomere in the cell. To directly test this hypothesis, we compared the senescence rates in independent *tlc1*-***Δ*** clones with isogenic clones lacking the last 15 kb of the 7L subtelomere. To obtain these strains, we generated diploids heterozygous for telomerase (*TLC1*/*tlc1*-***Δ***) and homozygous for subtelomere 7L (*7L-WT/7L-WT* or *7L-****Δ****15kb*/*7L-****Δ****15kb*) and derived spores to measure senescence rates (Fig. 4a). The subtelomeric region deleted in the *7L-Δ15kb* strain is the same as the 7L-CTL or −VST constructs^25,29^. No difference in growth rate was observed in *TLC1*-derived spores, while *tlc1-****Δ*** *7L-****Δ****15kb* strains senesced earlier than *tlc1-****Δ**** 7L-WT* (Fig. 4b,c). We concluded that the subtelomeric element of the 7L contains an essential region, able to promote viability in the absence of telomerase.

To determine the minimal genetic elements required for growth in the absence of telomerase, we generated similar strains in which only the terminal 8 kb of the 7L subtelomere, or an internal 8–14 kb were missing (Fig. 4a). Surprisingly, senescence was no longer accelerated in either deletion (Fig. 4d–g). Importantly, when growth of the *tlc1-****Δ*** *7L-****Δ****15kb* strain was compared with *tlc1*-***Δ*** *7L-****Δ****8 kb* or *tlc1-****Δ**** 7L-****Δ****8-14 kb* strain in senescence assays performed in parallel, a significant difference was measured in the three passages (Supplementary table 3, lanes corresponding to Fig. 4b–d and 4b*–f). Since *ADH4*, inactivated in *7L-Δ15 kb* constructs but kept intact in both *tlc1-****Δ*** *7L-****Δ****8 kb* or *tlc1-****Δ*** *7L-****Δ****8-14 kb* strains, was shown previously not to contribute to senescence^30^, we concluded that the deletion of 15 kb is likely unique in affecting growth in the absence of telomerase in that its effect could not be traced back to a smaller region.

### Silencing of internal genes does not explain the growth defect of telomerase-negative **Δ**15 kb strains

**Figure 5:**
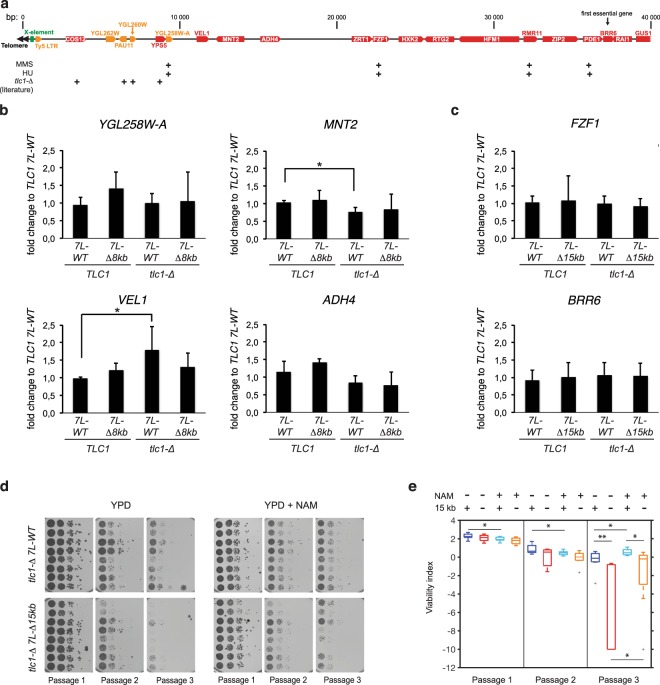
Chromatin is likely unchanged in strains lacking terminal 15 kb of 7L. (**a**) Genetic elements overexpressed upon methyl methanesulfonate (MMS) treatment, hydroxyurea (HU) treatment, or in *tlc1-****Δ*** are indicated with “+” signs, according to^39,44^. (**b**,**c**) mRNA levels of indicated coding sequences in indicated strains were assessed by qPCR, normalized by *ACT1* mRNA and by corresponding levels in *TLC1 7L-WT* strains. Error bars correspond to SD. *p-value < 0.05 obtained with one-tailed Student T-test. n ≥ 3. (**d**) Spores of the indicated genotype were spotted in parallel for three serial passages on YPD or YPD complemented with 5 mM Nicotinamide (NAM) and analyzed as in Fig. 2b,c (**e**). Adjusted P-values were obtained by the Wilcoxon rank-sum test with a false discovery rate correction *p-value < 0.05 and **p-value < 0.01. n = 8 for each strain set. See Supplementary Table 3 for detailed p-values.

One attractive hypothesis to explain the growth defect of the 15 kb deletion-containing strains is the silencing of telomere-proximal genes due to the telomere-position effect (TPE). For instance, genes that become essential upon telomerase inactivation and that are positioned near the telomere (inner to *ADH4*) may be silenced specifically in the *7L-Δ15 kb* construct. In favor of this hypothesis is the fact that the construct used to generate the *7L-****Δ****15kb* strains is the exact same than the one used to first demonstrate TPE and is known to induce a robust silencing^29^. If this were the case, coding sequences near the chromosome extremity should be silenced in the *7L-****Δ****15 kb* clones, and possibly in the *7L-****Δ****8 kb* cells, but at least one gene affected specifically in the *7L-Δ15 kb* truncation would be essential upon telomerase inactivation. We thus compared mRNA levels of coding sequences in the different constructs in the presence or absence of telomerase. We focused on genes that were found overexpressed in telomerase-negative conditions, upon genotoxic treatments and the first essential gene (Fig. 5a). This analysis showed that truncating 15 kb of the 7L subtelomere did not reduce substantially the steady state levels of any mRNA expressed close from the chromosome end (Fig. 5b,c). Interestingly, we found that *VEL1* and *MNT2* mRNA levels were slightly but reproducibly increased and decreased, respectively, in telomerase-negative cells, reminiscent of a potential co-regulation^31^. Yet, taken together, our results show no evidence for *de novo* heterochromatinization of telomere-proximal gene loci when subtelomere 7L is truncated.

TPE at 7L-**Δ**15 kb was shown to depend on the recruitment of the SIR complex composed of Sir2, Sir3 and Sir4 proteins^32^. More specifically, the activity of Sir2, which deacetylates histones in a nicotinamide adenine dinucleotide (NAD)-dependent manner, is key for the spreading of TPE. Thus, if growth defects in *tlc1-****Δ**** 7L-****Δ****15kb* cells, are due to silencing of telomere-proximal genes, then inhibiting Sir2 should lead to a suppression of the difference in growth between these two sets of strains. We thus tested senescence rates in the presence or absence of nicotinamide (NAM), a known inhibitor of Sir2 and other histone deacetylases^33^. We found that while NAM accelerated senescence in the first two passages as previously observed for early stages of senescence^34^, it decreased cell growth defects substantially in the third passage. Still, cells harboring the subtelomere *7L-****Δ****15kb* are significantly delayed in growth compared to *7L-WT* in NAM-containing media. We concluded that Sir2-dependent TPE is likely not involved in the growth defects observed in *tlc1-****Δ**** 7L-***Δ***15kb* cells. Taken together, our results suggest that the truncation of the 7L subtelomere, while affecting the expression of the telomere-proximal *URA3* marker^29^, does not affect the expression of more distal genes, especially a gene that would become essential in telomerase-negative cells.

## Discussion

By studying replicative senescence of telomerase-negative cells, in which we have modified the structure of only one of the telomeres, we show that removal of the last 15 kb of the corresponding subtelomere accelerates replicative senescence (Figs 2 and 4). This effect is abrogated when only the terminal 8 kb or internal 8–14 kb are deleted, suggesting that the 15 kb deletion affects in a unique manner the growth of telomerase-negative strains. More precisely, our results suggest that there is no single genetic element within the last 15 kb of the 7L that contributes to cell viability in a significant manner^30^. In particular, the X-element, a conserved ~475 bp region, present in all chromosome ends^35^, and absent in the *7L-****Δ****8* *kb* strains, does not contribute to cell viability when telomeres become short. Also, our data suggest that homologous recombination involving the subtelomere of the shortest telomere is not required to maintain cell growth (Fig. 3). Rather, we speculate that the TG_1-3_ repeats region of the shortest telomere might be directly involved in recombination or break-induced replication repair event, consistent with recombination mechanisms occurring in type II survivors^36,37^.

To explain the unique effect of the 15 kb deletion, we tested whether this truncation removed a buffer to protect essential internal genes from TPE, i. e., the silencing of genes due to heterochromatin nucleated at telomeres^38^. We found that Sir2 inhibition by NAM, which suppresses TPE and other SIR-dependent silencing^33,34^, does not suppress the poor growth due to the 15 kb deletion, when compared to cells containing the full length 7L subtelomere (Fig. 5d,e). Also, mRNA levels of *BRR6*, the first known essential gene in the subtelomere, are unchanged in cells containing 15 kb 7L-truncated construct (Fig. 5c). Likewise, mRNA levels of *FZF1*, shown to be upregulated in DNA damage conditions^39^, and much closer to the truncated DNA end, are unaffected. Besides, we observe that the 8 kb terminal truncation is irrelevant with respect to mRNA levels of genes that become closer to terminus in these conditions (Fig. 5b). Taken together, these observations are in accordance with the idea that the vast majority of subtelomeric genes are not subjected to Sir2-dependent TPE^24,40^. More specifically, TPE is likely not influencing internal genes that would become essential in the absence of telomerase.

To conclude, this work dissects the contribution of the subtelomere of the 7L chromosome end, either when it is engineered to be the shortest telomere in the cell or as a telomere of normal length, in the onset of senescence. Future work will certainly help to better delineate how subtelomeres affect cell fitness.

## Methods

### Yeast strains

All strains are derived from W303 and listed in Supplementary Table 1. yT674 and yT675 were generated by transformation of yT361 and yT362^26^, respectively, with a PCR cassette to delete *RAD51*^41^ using oligonucleotides oT323 and oT324. They were crossed with a spore of yT502 and yT503^41^ to obtain diploids yT502 and yT503 heterozygous for *RAD51*/*rad51::HIS3*. yT839 and yT840 were obtained by first generating a diploid of W303 in which *TLC1* was deleted^26^ to obtain yT789. yT789 was then transformed with pVII-L URA3-Tel^29^ to obtain yT790. This diploid was sporulated and dissected. Germinated spores were mated to obtain yT839 and yT840. yT1136 and yT1137 were obtained similarly, but plasmid pT58 was used instead of pVII-L URA3-Tel. pT58 was constructed by replacing the *ADH4* portion of pVII-L URA3-Tel by a PCR fragment containing a *YPS5* region (obtained using oligonucleotides oT1152 and oT1153). yT839 was further transformed with a PCR cassette to delete the 8–14 kb region using oligonucleotides oT1096 and oT1099 and pFA6a-His3MX6 to obtain yT1376 according to^41^. This diploid was sporulated and spores were crossed to obtain yT1377 and yT1378.

### Senescence assay upon mass germination

Senescence assay of strains containing the shortening system was performed as described^17^. Briefly, diploids (i) carrying one allele of the telomerase RNA template gene *TLC1* replaced by the nourseothricin gene resistance marker under the control of a *Mat* α-specific promoter, (ii) homozygous for the telomeric constructs and (iii) carrying the *FLP1* gene under the control of the *GAL10* promoter, were mass sporulated and germinated in rich medium containing galactose for 6–9 hours. To control *FLP1* induction, germination of the same sporulation mixture was also performed in glucose–containing media. Telomerase-negative spores were selected by plating the germination mixture on Nourseothricin-containing media. After 2 days, colonies were genotyped, verified for the loss of the *URA3* marker and for telomere length by telomere-PCR^42^ using oligonucleotides listed in Supplementary Table 2. Remaining cells were then resuspended, adjusted to the same concentration, serially diluted 10 fold and then spotted on a solid media, followed by a two days incubation at 30 °C (passage 1). This procedure was repeated every two days using mixed cells from the most concentrated spot until complete loss of viability, typically 2 times (passage 2 and passage 3). Plates were scanned with an Epson Perfection V750 Pro and analyzed as described^14^ to obtain the viability plots. Supplementary Table 3 details the corresponding statistical analysis.

### Senescence assay upon tetrad dissection

Similarly to above, except that tetrads obtained from sporulation of heterozygous *TLC1/tlc1-****Δ*** diploids were manually dissected (MSM 400, Singer Instruments, UK) on YPD plates and left to grow at 30 °C for 2 days. After genotyping of resulting colonies, spot assays were performed as described above. Where indicated, Nicotinamide (NAM, Sigma-Adrich N0636) was added to YPD plates at 5 mM.

#### Telomere length analysis

Southern blots and telomere-PCR were performed as described^2,26,42^. For telomere-PCR, in brief, genomic DNA was C-tailed by Terminal Transferase (New England Biolabs) and then amplified by Taq polymerase with a poly-G primer and primers listed in Supplementary Table 2 targeting specifically the Y’-containing telomeres, 7L or 6R telomeres. PCR products were electrophoresed and stained with Ethidium Bromide. Mean telomere length was estimated using the ImageLab software (Biorad).

#### Chromatin immunoprecipitation

The protocol described previously^17^ was slightly modified. Briefly, after germination in galactose-containing media, cells were plated on galactose- and nourseothricine-containing solid media and grown for two days at 30 °C. This allowed excision of the *URA3*-containing circle with more than 95% efficiency. Colonies were then pooled and grown to OD_600nm_ = 0.8 and prepared for chromatin immunoprecipitation as reported^17^ using oligonucleotides listed in Supplementary Table 2.

#### RNA extraction and RT-qPCR

RNA extraction was adapted^43^. 2.10^8^ exponentially growing cells were pelleted and resuspended in 400 µL of 50 mM CH_3_COONa, pH 5.3; 10 mM EDTA. 40 µL of 20% SDS was added, followed by 440 µL of acidic phenol preheated to 65 °C. The samples were vigorous mixed, incubated at 65 °C for 10 minutes, frozen in N_2_(l) and centrifuged at ~15000 g for 15 min at room temperature. The upper aqueous phase was recovered and mixed with one volume of phenol/chloroform, followed by a similar centrifugation for 5 min. The upper aqueous phase was precipitated o/n at −20 °C by adding 1/10th volume of 3 M CH_3_COONa, pH 5.3 and 2 volumes of 100% Ethanol. Pellets were recovered by a 25 min centrifugation at ~15 000 g at 4 °C, washed with 300 µL of 70% EtOH, vacuum dried for 10 minutes, resuspended in 50 µL of nuclease-free water (Millipore, ref. # H2OMB0106) and quantified using Nanodrop 2000 (Thermo Scientific). 30 µg of RNAs were adjusted to 100 µL of water containing 10 µL of Buffer RDD and 2.5 µL of DNAse (Qiagen ref. # 79254). After 1 hour of incubation at room temperature, an additional 1 µL of DNAse was added and the samples were further incubated 30 minutes. RNA was further cleaned up by phenol-chloroform extraction, precipitated, washed in 70% Ethanol, vacuum dried, resuspended in 100 µL nuclease-free water and purified using the RNAEasy Min Elute Kit (Qiagen ref. # 74204). DNAse treatment was repeated and RNA was finally recovered in 15 µL of nuclease-free water and quantified as above.

Reverse transcription (RT) was performed on 3 µg of purified RNAs using the Superscript IV first-strand cDNA synthesis kit (Thermofischer ref. # 18091050) and an oligo-(dT)_20_ primer according to the manufacturer’s protocol, including an RNAseH (2U/µl) treatment. As a control the same reaction was performed on all samples substituting the SSIV reverse transcriptase with 1 µL of nuclease-free water (−RT control). RT reactions were diluted 4 fold and 2 µL of these dilutions were used for two technical replicates of qPCR using the iTaq qPCR kit (BioRad, ref. # 1725124) and BioRad system (CFX96 C1000 touch). Primers used for the qPCR analysis are listed in Supplemental Table 2 and their efficiency was similar (oT794&oT795: 100,9%; oT1611/1612: 99,5%; oT1605/1606: 101,7%; oT1607/1608: 98%; oT1609/1610: 94,8%; oT0831/oT0832: 100%; oT1656/1657: 99,7%). Quantification of the qPCR data was done with the ΔΔ method using *ACT1* mRNA as reference.

## Supplementary information

Supplementary Information
