# Supplementary material for: A subtelomeric region affects telomerase-negative replicative senescence in Saccharomyces cerevisiae
Source: Sci Rep. 2019 Feb 12;9:1845. doi: 10.1038/s41598-018-38000-9 (PMC6372760; doi:10.1038/s41598-018-38000-9)
Supplement: Supplementary file 1 — Supplementary Information [file 41598_2018_38000_MOESM1_ESM.pdf]

Supplementary Information for:

A subtelomeric region affects telomerase-negative replicative senescence in *Saccharomyces cerevisiae*

Pascale Jolivet[#], Kamar Serhal[#], Marco Graf, Stephan Eberhard, Zhou Xu, Brian Luke and Maria Teresa Teixeira[*]

Supplementary Table 1:

Yeast strains used in this study

All strains are in the W303 background. The strains were constructed for this work, except those labelled with *, which were described previously[1-3].

Strain	Genotype
yT136*	Mata/α cir° ura3-1/ura3-1 trp1-1/trp1-1 his3-11,15/his3-11,15 can1-100/can1-100 ade2-1/ade2-1 leu2-3,112::Gal-FLP1-LEU2/leu2-3,112::Gal-FLP1-LEU2 rad5-535/rad5-535 tlc1::PrαNat/TLC1 telVIIL::FRT-URA3-FRT-tel/telVIIL::FRT-URA3-FRT-tel
yT138*	Mata/α cir° ura3-1/ura3-1 trp1-1/trp1-1 his3-11,15/his3-11,15 can1-100/can1-100 ade2-1/ade2-1 leu2-3,112::Gal-FLP1-LEU2/leu2-3,112::Gal-FLP1-LEU2 rad5-535/rad5-535 tlc1::PrαNat/TLC1 telVIIL::FRT-URA3-[tel600]-FRT-tel/telVIIL::FRT-URA3-[tel600]-FRT-tel
yT347*	Mata/α cir° ura3-1/ura3-1 trp1-1/trp1-1 his3-11,15/his3-11,15 can1-100/can1-100 ade2-1/ade2-1 leu2-3,112::Gal-Flp1-Leu2/leu2-3,112::Gal-Flp1-Leu2 tlc1::PrαNat/TLC1 telVIIL::FRT-URA3-FRT/telVIIL::FRT-URA3-FRT-tel pCen-ARS-TRP1-TLC1 rad51::HIS3/RAD51
yT348*	Mata/α cir° ura3-1/ura3-1 trp1-1/trp1-1 his3-11,15/his3-11,15 can1-100/can1-100 ade2-1/ade2-1 leu2-3,112::Gal-Flp1-Leu2/leu2-3,112::Gal-Flp1-Leu2 tlc1::PrαNat/TLC1 telVIIL::FRT-URA3-[tel600]-FRT/telVIIL::FRT-URA3-[tel600]-FRT-tel pCen-ARS-TRP1-TLC1 rad51::HIS3/RAD51
yT502*	Mata/α cir° ura3-1/ura3-1 trp1-1/trp1-1 his3-11,15/his3-11,15 can1-100/can1-100 ade2-1/ade2-1 leu2-3,112::Gal-FLP1-LEU2/leu2-3,112::Gal-FLP1-LEU2 rad5-535/rad5-535 tlc1::PrαNat/TLC1 telVIR::FRT-URA3-FRT-tel/telVIR::FRT-URA3-FRT-tel
yT503*	Mata/α cir° ura3-1/ura3-1 trp1-1/trp1-1 his3-11,15/his3-11,15 can1-100/can1-100 ade2-1/ade2-1 leu2-3,112::Gal-FLP1-LEU2/leu2-3,112::Gal-FLP1-LEU2 rad5-535/rad5-535 tlc1::PrαNat/TLC1 telVIR::FRT-URA3-[tel600]-FRT-tel/telVIR::FRT-URA3-[tel600]-FRT-tel
yT674	Mata cir° ura3-1 trp1-1 his3-11,15 can1-100 ade2-1 leu2-3,112::Gal-FLP1-LEU2 rad5-535 TLC1 telVIR::FRT-URA3-FRT-tel rad51::HIS3
yT675	Mata cir° ura3-1 trp1-1 his3-11,15 can1-100 ade2-1 leu2-3,112::Gal-FLP1-LEU2 rad5-535 TLC1 telVIR::FRT-URA3-[tel600]-FRT-tel rad51::HIS3
yT839	Mata/α cir° ura3-1/ura3-1 trp1-1/trp1-1 leu2-3,112/leu2-3,112 his3-11,15/his3-11,15 ade2-1/ade2-1, can1-100/can1-100 TLC1/tlc1::KanMX6 TELVIIL/TELVIIL
yT840	Mata/α cir° ura3-1/ura3-1 trp1-1/trp1-1 leu2-3,112/leu2-3,112 his3-11,15/his3-11,15 ade2-1/ade2-1, can1-100/can1-100 TLC1/tlc1::KanMX6 telVIIL-adh4::URA3-tel/telVIIL-adh4::URA3-tel
yT1136	Mata/α cir° ura3-1/ura3-1 trp1-1/trp1-1 leu2-3,112/leu2-3,112 his3-11,15/his3-11,15 ade2-1/ade2-1, can1-100/can1-100 TLC1/tlc1::KanMX6 TELVIIL/TELVIIL
yT1137	Mata/α cir° ura3-1/ura3-1 trp1-1/trp1-1 leu2-3,112/leu2-3,112 his3-11,15/his3-11,15 ade2-1/ade2-1, can1-100/can1-100 TLC1/tlc1::KanMX6 telVIIL-yps5::URA3-tel/telVIIL-yps5::URA3-tel
yT1376	Mata/α cir° ura3-1/ura3-1 trp1-1/trp1-1 leu2-3,112/leu2-3,112 his3-11,15/his3-11,15 ade2-1/ade2-1, can1-100/can1-100 TLC1/tlc1::KanMX6 TELVIIL/telVIIL 8-14kb::HIS

yT1377	Mata/α cir° ura3-1/ura3-1 trp1-1/trp1-1 leu2-3,112/leu2-3,112 his3-11,15/his3-11,15 ade2-1/ade2-1, can1-100/can1-100 TLC1/tlc1::KanMX6 TELVIIL/TELVIIL
yT1378	Mata/α cir° ura3-1/ura3-1 trp1-1/trp1-1 leu2-3,112/leu2-3,112 his3-11,15/his3-11,15 ade2-1/ade2-1, can1-100/can1-100 TLC1/tlc1::KanMX6 telVIIL 8-14kb::HIS/telVIIL 8-14kb::HIS

Supplementary Table 2:

Oligonucleotides used in this study (matching sequences of strain W303 - Sanger Institute database).

Name	Use	Sequence 5'-3'
oT323	RAD51 F1	TTCTTCTATCTTCCGTAGTTTCCATATACTAGTAGTTGAGTGTAGCGACAcggatccccgggttaattaa
oT324	RAD51 R1	GGATGGAAATGAAGATAAAAATGTACGGAACGCAACCTAAGAAAAAGAGGgaattcgagctcgtttaaac
169	Telomere-PCR	CGGGATCCGGGGGGGGGGGGGGGGGG
oT182	Telomere-PCR Y'	TACATGAGGGCTATTTAGGGCTATTTAGGG
oT156	Telomere-PCR VIIL	TTGTTGAAAGCCATACCTGCC
oT531	Telomere-PCR VIR	AGGACTGGGTCATGGGGCGC
oT794	qPCR ACTIN F	CCCAGGTATTGCCGAAAGAATGC
oT795	qPCR ACTIN R	TTTGTTGGAAGGTAGTCAAAGAAGCC
oT831	qPCR FZF1 F	TGATAGCACTACGGCACAAACCC
oT832	qPCR FZF1 R	TTTGCACCGGTTATCCGACCAG
oT1605	qPCR VEL1 F	AGGGCCTACATGGACCTCATTG
oT1606	qPCR VEL1 R	AGCATCACCAGCGCTTTCAGTC
oT1607	qPCR MNT2 F	TCCAGTGGTTGCACAGGTTCTG
oT1608	qPCR MNT2 R	CCAGGCCGCTTTCCAAATGATG
oT1609	qPCR ADH4 F	ACTCCAGCTGTTGCTGTCAACG
oT1610	qPCR ADH4 R	GCAGTCAAAGCAGGTGGCAAAC
oT1611	qPCR YGL258W-A F	GGGAGCATCTTATTCGGTGCAGTC
oT1612	qPCR YGL258W-A R	ACGCATAGGATGTGTGCAGAGC
oT1656	qPCR BBR6 F	AGACAGCCTGATGGCATACTTGC
oT1657	qPCR BBR6 R	AGCATCTTGCGGATGTTCACCTTC
oT1096	YPS5 F1	TGGGACAGGGTTTTGCATCGTTCTGTCTTGTTCTCTTTTTTCATTGGTTGATCAGTTGTTcggatccccgggttaattaa
oT1099	MNT2 R1	CGCATGAGTGTTTGCAAATCCGCAGTGACAGGACAAAAGTAGCCCCAATAGTATTTATCGgaattcgagctcgtttaaac

Supplementary Table 3:

p-values for the spot assays determined by pairwise Wilcoxon rank sum test with a false discovery rate adjustment

Experiment	Genotype 1	Genotype 2	Passage	p- value

Fig 2c	Control 7L	VST 7L	1	0,0255
	Control 7L	VST 7L	2	0,00005
	Control 7L	VST 7L	3	0,08568
	Control 6R	VST 6R	1	0,0006
	Control 6R	VST 6R	2	0,0078
	Control 6R	VST 6R	3	0,0098
	Control 7L	Control 6R	1	0,00042
	Control 7L	Control 6R	2	0,00036
	Control 7L	Control 6R	3	0,000008
	VST 7L	VST 6R	1	0,5935
	VST 7L	VST 6R	2	0,000008
	VST 7L	VST 6R	3	0,000007
Fig 3a	VST 7L RAD51	VST 7L rad51	1	0,0081
	VST 7L RAD51	VST 7L rad51	2	0,0069
	VST 7L RAD51	VST 7L rad51	3	0,1550
	VST 6R RAD51	VST 6R rad51	1	0,0317
	VST 6R RAD51	VST 6R rad51	2	0,0317
	VST 6R RAD51	VST 6R rad51	3	0,4206
	VST 7L RAD51	VST 6R RAD51	1	0,755
	VST 7L RAD51	VST 6R RAD51	2	0,0732
	VST 7L RAD51	VST 6R RAD51	3	0,005
	VST 7L rad51	VST 6R rad51	1	0,329
	VST 7L rad51	VST 6R rad51	2	0,0389
	VST 7L rad51	VST 6R rad51	3	0,2207
Fig 4b	TLC1+ 7L WT	TLC1+ 7L Δ 15 kb	1	0,2344
	TLC1+ 7L WT	TLC1+ 7L Δ 15 kb	2	0,0498
	TLC1+ 7L WT	TLC1+ 7L Δ 15 kb	3	0,1948
	TLC1- 7L WT	TLC1- 7L Δ 15 kb	1	0,0281
	TLC1- 7L WT	TLC1- 7L Δ 15 kb	2	0,0029
	TLC1- 7L WT	TLC1- 7L Δ 15 kb	3	0,0065
	TLC1+ 7L WT	TLC1- 7L WT	1	0,8784
	TLC1+ 7L WT	TLC1- 7L WT	2	0,0003
	TLC1+ 7L WT	TLC1- 7L WT	3	0,0001
	TLC1+ 7L Δ 15 kb	TLC1- 7L Δ 15 kb	1	0,0649
	TLC1+ 7L Δ 15 kb	TLC1- 7L Δ 15 kb	2	0,0001
	TLC1+ 7L Δ 15 kb	TLC1- 7L Δ 15 kb	3	0,0001
Fig 4d	TLC1+ 7L WT	TLC1+ 7L Δ 8 kb	1	0,4418
	TLC1+ 7L WT	TLC1+ 7L Δ 8 kb	2	0,0498
	TLC1+ 7L WT	TLC1+ 7L Δ 8 kb	3	0,0379
	TLC1- 7L WT	TLC1- 7L Δ 8 kb	1	0,7209
	TLC1- 7L WT	TLC1- 7L Δ 8 kb	2	0,8784
	TLC1- 7L WT	TLC1- 7L Δ 8 kb	3	0,7209
	TLC1+ 7L WT	TLC1- 7L WT	1	0,0029
	TLC1+ 7L WT	TLC1- 7L WT	2	0,0001
	TLC1+ 7L WT	TLC1- 7L WT	3	0,0001
	TLC1+ 7L Δ 8 kb	TLC1- 7L Δ 8 kb	1	0,0046
	TLC1+ 7L Δ 8 kb	TLC1- 7L Δ 8 kb	2	0,0001
	TLC1+ 7L Δ 8 kb	TLC1- 7L Δ 8 kb	3	0,0001
Fig 4b-4d	TLC1- 7L Δ 8 kb	TLC1- 7L Δ 15 kb	1	0,0029
	TLC1- 7L Δ 8 kb	TLC1- 7L Δ 15 kb	2	0,0069
	TLC1- 7L Δ 8 kb	TLC1- 7L Δ 15 kb	3	0,0133

Fig 4f	TLC1+ 7L WT	TLC1+ 7L Δ 8-14 kb	1	0,0649
	TLC1+ 7L WT	TLC1+ 7L Δ 8-14 kb	2	0,0147
	TLC1+ 7L WT	TLC1+ 7L Δ 8-14 kb	3	0,3282
	TLC1- 7L WT	TLC1- 7L Δ 8-14 kb	1	0,8784
	TLC1- 7L WT	TLC1- 7L Δ 8-14 kb	2	0,8784
	TLC1- 7L WT	TLC1- 7L Δ 8-14 kb	3	0,2344
	TLC1+ 7L WT	TLC1- 7L WT	1	0,0104
	TLC1+ 7L WT	TLC1- 7L WT	2	0,0001
	TLC1+ 7L WT	TLC1- 7L WT	3	0,0001
	TLC1+ 7L Δ 8-14 kb	TLC1- 7L Δ 8-14 kb	1	0,0498
	TLC1+ 7L Δ 8-14 kb	TLC1- 7L Δ 8-14 kb	2	0,0001
	TLC1+ 7L Δ 8-14 kb	TLC1- 7L Δ 8-14 kb	3	0,0001
Fig 5c	TLC1- 7L WT	TLC1- 7L Δ 15 kb	1	0,2786
	TLC1- 7L WT	TLC1- 7L Δ 15 kb	2	0,2786
	TLC1- 7L WT	TLC1- 7L Δ 15 kb	3	0,0010
	TLC1- 7L WT	TLC1- 7L WT NAM	1	0,0379
	TLC1- 7L WT	TLC1- 7L WT NAM	2	0,0498
	TLC1- 7L WT	TLC1- 7L WT NAM	3	0,0379
	TLC1- 7L Δ 15 kb	TLC1- 7L Δ 15 kb NAM	1	0,1303
	TLC1- 7L Δ 15 kb	TLC1- 7L Δ 15 kb NAM	2	0,2344
	TLC1- 7L Δ 15 kb	TLC1- 7L Δ 15 kb NAM	3	0,0270
	TLC1- 7L WT NAM	TLC1- 7L Δ 15 kb NAM	1	0,3282
	TLC1- 7L WT NAM	TLC1- 7L Δ 15 kb NAM	2	0,1605
	TLC1- 7L WT NAM	TLC1- 7L Δ 15 kb NAM	3	0,0104
Fig 4b*-4f	TLC1- 7L Δ 8-14 kb	TLC1- 7L Δ 15 kb	1	0,0093
	TLC1- 7L Δ 8-14 kb	TLC1- 7L Δ 15 kb	2	0,0093
	TLC1- 7L Δ 8-14 kb	TLC1- 7L Δ 15 kb	3	0,0012

**tlc1-Δ 7L-Δ 15kb* clones as shown in Figure 4b were respotted at the same time than *tlc1-Δ 7L-Δ 8-14 kb* clones shown in Figure 4b to allow pairwise comparison.

Supplementary Figure 1

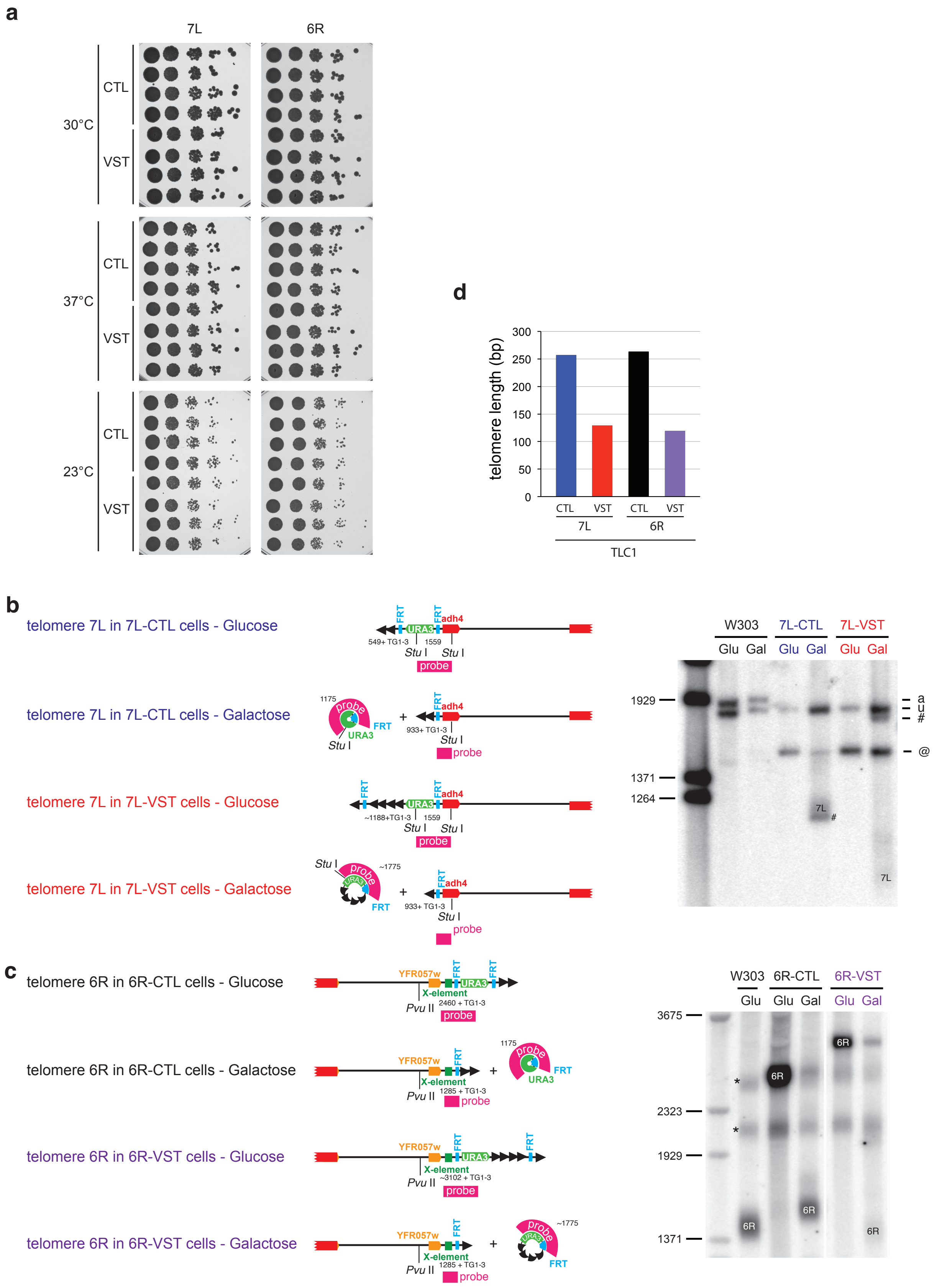

e

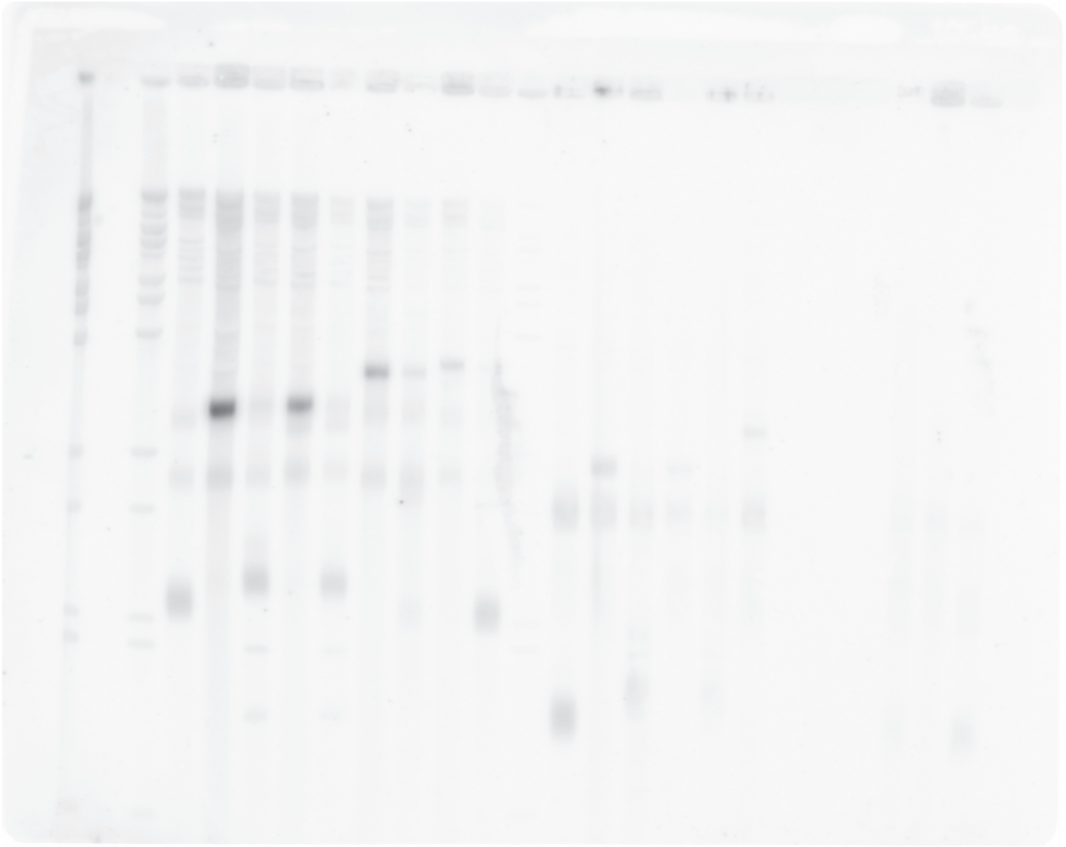

f

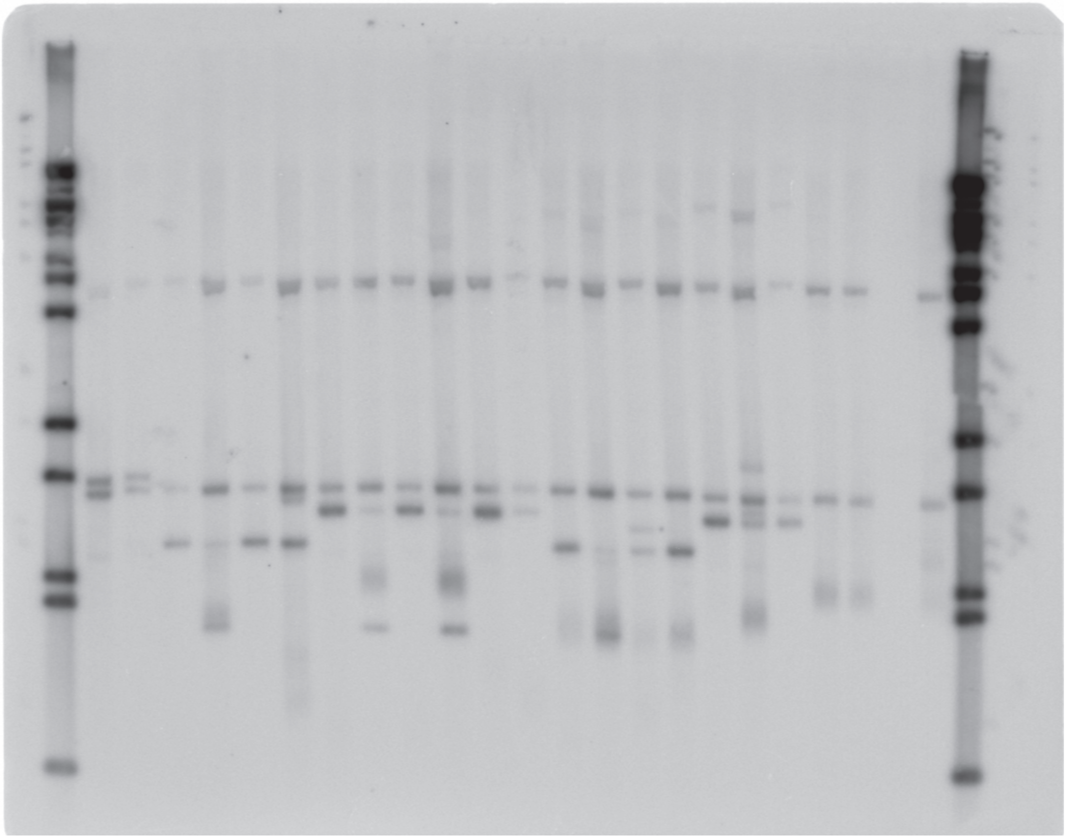

(a) Serial spot assay of telomerase-positive cells containing the constructs shown in Figure 1B, C, E, F (7L-CTL: lev187; 7L-VST: lev220; 6R-CTL: yT361; 6R-VST: yT362 [2,4]). 10-fold serial dilutions of 4 independent clones were spotted on minimal media and then incubated for 2 days at 30°C, 37°C or 23°C. (b-c) Schematic representation of Telomere Restricted Fragment (TRF) analysis and corresponding Southern blots of telomere 7L (b) and 6R (c) of strains labeled as in (a) and Figure 1. Southern blot in (c) is the exact same Figure than Figure S2G from[4], where these set of strains were first described. Cells were cultivated for four hours in a rich medium containing either glucose or galactose, to induce shortening, followed by DNA extraction and DNA restrictive digestion by *Stu* I (B) or *Pvu* II (c). u: internal *ADH4* locus; a: internal *ura3* locus ; #: excised URA3-containing circle (linearized); @: fragment of the 7L when excision did not occur; *: corresponds to cross hybridization to other X elements in the genome; 7L: 7L TRF; 6R: 6R TRF. (d) Telomere lengths of 7L and 6R telomeres in CTL or VST strains estimated from by Southern Blot after *FLP1* induction before telomerase removal shown in B-C. (e-f) Original images of the Southern blots shown in (b-c). Lanes shown in b) correspond to lanes 3-4 and 9-10 of (e). Lanes shown in (c) correspond to lanes 1-7 of (f).

References

1 Abdallah, P. *et al.* A two-step model for senescence triggered by a single critically short telomere. *Nat Cell Biol* **11**, 988-993 (2009).

2 Marcand, S., Brevet, V. & Gilson, E. Progressive cis-inhibition of telomerase upon telomere elongation. *EMBO J.* **18**, 3509-3519 (1999).

3 Fallet, E. *et al.* Length-dependent processing of telomeres in the absence of telomerase. *Nucleic Acids Res* **42**, 3648-3665 (2014).

4 Graf, M. *et al.* Telomere Length Determines TERRA and R-Loop Regulation through the Cell Cycle. *Cell* **170**, 72-85 (2017).
